# Diurnal raptors at rescue centres in the Czech Republic: Reasons for admission, outcomes, and length of stay

**DOI:** 10.1371/journal.pone.0279501

**Published:** 2022-12-30

**Authors:** Gabriela Kadlecova, Eva Voslarova, Vladimir Vecerek

**Affiliations:** Department of Animal Protection and Welfare and Veterinary Public Health, Faculty of Veterinary Hygiene and Ecology, University of Veterinary Sciences Brno, Brno, Czech Republic; UFERSA: Universidade Federal Rural do Semi-Arido, BRAZIL

## Abstract

Rescue centres play an important role in the protection of raptors living in the wild by caring for injured or debilitated animals and abandoned young with the aim of returning them to the wild. A total of 22,538 raptors were admitted to 34 rescue centres in the Czech Republic in the years 2010–2019, with an increasing trend during the monitored period (rSp = 0.7333, p < 0.05). The most frequent reasons for their admission were other injuries and fractures (26.52%), the admission of young (22.98%), and the admission of raptors injured by electric shock injuries (20.51%). It proved possible to release 42.45% of admitted raptors back into the wild, the majority of which (91.05%) were released using the hard-release method. Foster parents were used in 1% of cases and a replacement nest in 0.2% of cases involving the rearing of young. In spite of all the care provided at rescue centres, a total of 39.97% of raptors admitted either died or had to be euthanized. Among them, most raptors were euthanized or died due to injuries caused by collision with a vehicle, electric shock injuries, and other injuries. This generally occurred shortly after admission (a median of two days). The importance of the work of rescue centres lies not merely in returning injured raptors back into the wild (which proves possible in around half of all cases), but also in obtaining information about the factors endangering raptors in the wild and contributing toward a decline in their populations. The findings provide information about human-wildlife interactions in the Czech Republic and their implications for conservation as well as on the effectiveness of rescue centres to successfully treat and subsequently release raptors back into the wild.

## Introduction

Raptors are a group of birds adapted to hunting prey using their excellent eyesight [1, 2] and specialised hunting techniques [3]. Although a large proportion of their attacks are unsuccessful [4], their presence in the landscape is responsible for reducing the populations of their prey, such as rodents [5]. The potential role played by raptors as effective means in the fight against the spread of certain zoonoses is also associated with this [6]. Raptor populations in a given region are not stable [7] and are often influenced by the characteristics of the specific area and a number of other demands. A large number of raptor species are, nevertheless, demonstrably endangered today [8]. The Red List of Vertebrates of the Czech Republic of 2017 ranks the red kite (*Milvus milvus*) and the white-tailed eagle (*Haliaeetus albicilla*), for example, among critically endangered raptors in the Czech Republic, while the red-footed falcon (*Falco vespertinus*) is considered completely extinct [9].

Anthropogenic activity endangers raptors in many ways. Monocultures and a low level of environmental diversity have a negative effect on raptor populations [10], since monocultures do not provide opportunities to observe the landscape and search for potential prey. Power lines and high-voltage cables, often mentioned in connection with the mortality of raptors, cause great losses to raptor populations in many parts of the world [11, 12]. The mortality of various raptor species is also linked to the design of power lines and includes also endangered species [13]. Raptors can be injured or killed by vehicles [14] and also suffer from poisoning, for example, botulinum toxin poisoning [15], and the risk of secondary poisoning with, for example, rodenticides designed to exterminate rodents damaging to agricultural crops [16, 17]. Although the benefits of raptors to the natural world and their place in the landscape go without saying to many, some members of the public, gamekeepers, and pigeon fanciers believe raptors to be pests that cause damage by hunting pigeons, domestic poultry, and small game animals. Raptors are, for this reason, deliberately poisoned by people in the wild [18]. Carbofuran, for example, a substance that has already been banned, is still used illegally and placed in the wild in baits. The raptor either eats the bait directly or (in cases of secondary poisoning) eats a rodent killed or debilitated by poisoning. In spite of great efforts, attempts to find the perpetrators are often unsuccessful and poisoning is ongoing [19, 20].

Birds including raptors make up a large proportion of animals admitted to rescue centres [21], many of them being admitted due to anthropogenic causes [22]. The main aim of rescue centres is to mitigate the impact of human activities on wildlife species. In the Czech Republic, there is a network of rescue centres that care for wild animals (including raptors) that are not capable of surviving in the wild, primarily as a result of human activities. Raptors that are injured or poisoned, that are apathetic or display other signs of abnormal behaviour, or that are orphaned as young are admitted to rescue centres, where they are provided with appropriate care and subsequently released back into the wild after their recovery. This study aimed to assess the number of diurnal raptors admitted to rescue centres in the Czech Republic in the period 2010–2019, to assess the causes of their admission to rescue centres and their outcomes, and to evaluate the length of time they spent at these rescue centres.

## Materials and methods

Data were obtained from the database of the Ministry of the Environment including data on all diurnal raptors admitted to the 34 rescue centres in the Czech Republic belonging to the National Network of Rescue Centres from 1 January 2010 to 31 December 2019. In addition to the numbers of raptors, this database also includes the reasons for their admission and their outcomes and the dates on which animals were admitted to rescue centres. The raptors were admitted to the rescue centres for health reasons and were treated in accordance with the Czech legislation governing the operation of rescue centres and veterinary care, including the Act on the Protection of Animals against Cruelty.

The total number of diurnal raptors admitted in individual years of the monitored period and the number of birds of individual raptor species were assessed. For the purposes of analysis, those species of which less than fifty individuals (<0.01% of the total) were admitted during the entire monitored period were assigned collectively to the group “Other”. Conservation status was checked for each species both in the Czech law and IUCN Red List to provide additional information on admitted raptors. The numbers of raptors were also assessed according to the reasons for their admission to rescue centres (Table 1) and their outcomes (Table 2). Trends in the numbers of raptors admitted due to the most common reasons for admission (transport–road and rail, electric shock injuries, young and other injuries) in the most frequently admitted species during the monitored period were also evaluated as well as the proportion of raptors in these groups that died or had to be euthanized.

**Table 1:** Reasons for the admission of raptors to rescue centres in the Czech Republic used by the National Network of Rescue Centres.

Reason for admission	Description
**Electric shock injuries**	Found with burns from high-voltage cables or lying in the vicinity of power lines.
**Other injuries**	Found with fractures, bleeding injuries, gunshot wounds, bite wounds or paralysis.
**Young**	Young found in abandoned nests, fallen from nests, debilitated young.
**Transport–road, rail**	Found in the vicinity of roads or rail lines showing signs of a collision with a road or rail vehicle.
**Falls into chimneys, etc.**	Found after falling into a chimney or another aperture from which they were unable to escape on their own.
**Exhaustion and infection**	Found with signs of infection (including parasitic), exhaustion caused by starvation, apathetic raptors.
**Inadvertent or unnecessary capture**	Raptors without obvious signs of injury or caught inadvertently and brought to rescue centres.
**Trained raptors**	Tagged raptors that have escaped from their owners and been identified.
**Trapped or entangled**	Raptors that have become trapped or entangled in manmade materials from which they could not escape or that have remained on their bodies and impeded their movement.
**Poisoned**	Raptors with signs of poisoning without signs of injury (e.g. signs of Carbofuran poisoning).
**Hatchling**	Young hatched at a rescue centre.
**Other reasons**	Reasons other than the above (e.g. transfer to a safe place, weather conditions, found flying inside the building, damaged feathers due to unknown reason)

**Table 2:** Outcomes for raptors at rescue centres in the Czech Republic.

Outcome	Description
**Hard release**	Raptors released directly into the wild.
**Soft release**	Raptors released by a process of gradually accustoming to a new environment before releasing them into it (the possibility of returning to an enclosure, providing food at an accessible place, etc.).
**Adoption**	Young raptors placed in a different nest and accepted by a foster mother bird or raised by a pair in permanent captivity at a rescue centre.
**The use of a replacement nest**	Raptors moved to a replacement nest following their original nest being destroyed or made inaccessible.
**Death, euthanasia**	Raptors dying or being euthanized for medical reasons by a veterinary surgeon at a rescue centre.
**Released without going into captivity**	Raptors whose problem is resolved at the finding site, e.g. caught up in a net or other manmade material and released immediately without being housed at a rescue centre.
**Under continuing treatment**	Raptors still being treated at a rescue centre at the time of completion of the study.
**Returned to owner**	Raptors escaping from captivity and returned to their owners after being captured.
**Permanent captivity**	Raptors that could not be returned to the wild.
**Other**	Other outcomes (e.g. raptors that have escaped from a rescue centre during handling).

The length of stay was calculated for raptors for which an exact date of admission to a rescue centre and a date when their stay in the rescue centre was terminated were recorded. The length of stay was compared in relation to the individual outcomes of these raptors. For the purposes of evaluating their length of stay, raptors were divided into the following outcome categories: hard release, soft release, adoption, death or euthanasia, returned to a nest/replacement nest, and returned to the owner.

The program UNISTAT 6.5 for Excel (Unistat Ltd., London, UK) was used for statistical processing. To assess trends in the number of raptors admitted in the years 2010 to 2019, the Spearman’s correlation coefficient was used to determine a rank correlation coefficient. Differences in frequencies in individual studied groups were evaluated by means of a Chi-square test with Yates correction using a 2x2 contingency table methodology. Kruskal-Wallis ANOVA (and subsequent multiple comparisons for t-distributions) was used for statistical comparison of the length of stay between the individual categories according to the method of termination of the stay at rescue centres. A value of p < 0.05 was determined to be statistically significant.

## Results

**Fig 1:**
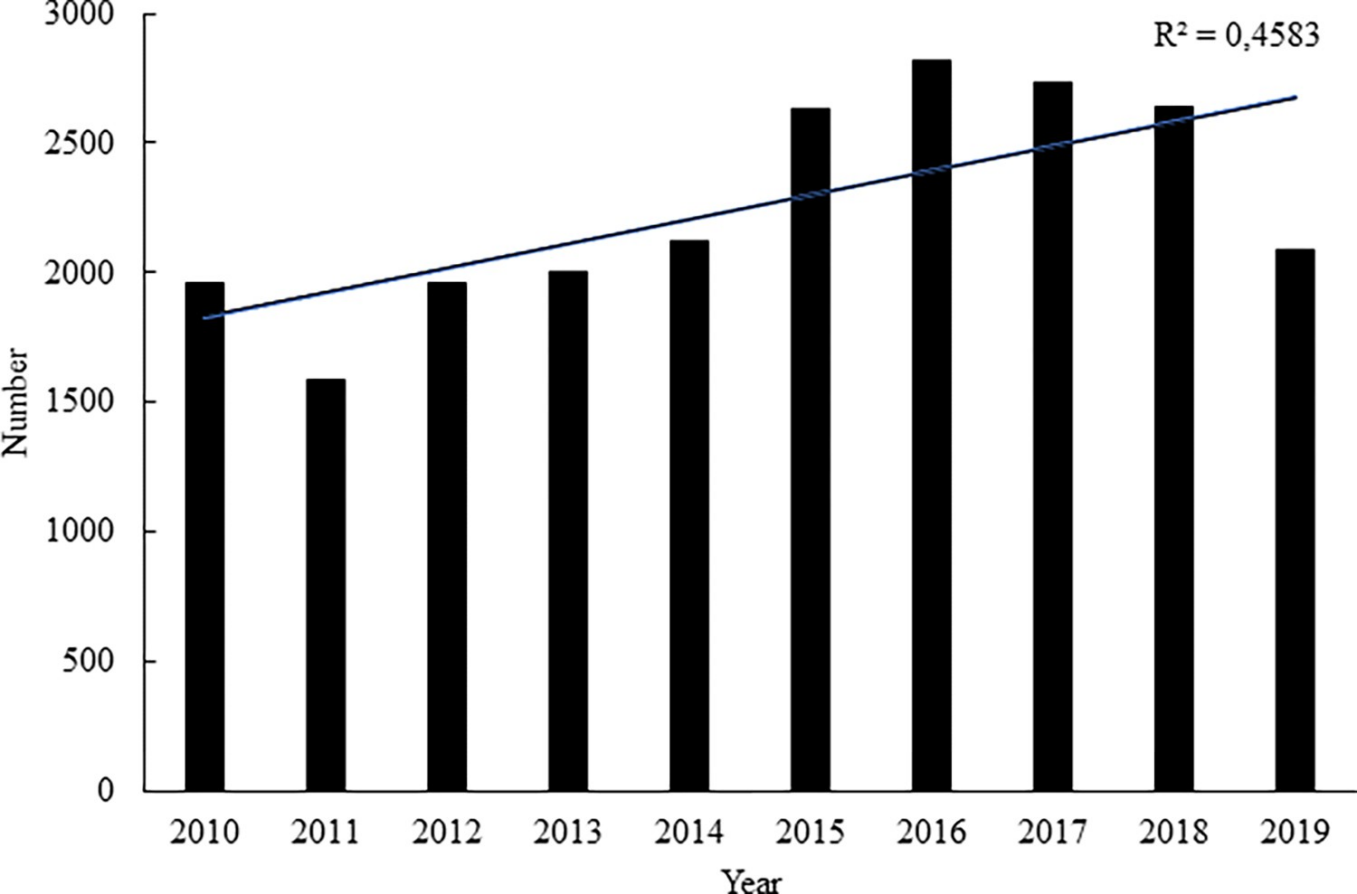
Number of raptors admitted to rescue centres in the Czech Republic in the individual years of the monitored period.

A total of 22,538 raptors were admitted to 34 rescue centres in the period from 2010 to 2019, with an increasing trend in the number of raptors admitted confirmed during the monitored period (rSp = 0.7333, p < 0.05) (Fig 1).

Raptors admitted to rescue centres in the monitored period were of 27 species. In 11 species, the number of individuals exceeded 50 (Table 3). The species admitted to rescue centres most often were the common kestrel (57.76%), the common buzzard (25.54%), and the Eurasian sparrowhawk (8.42%). Other species accounted for less than 3% of the raptors admitted.

**Table 3:** Number of raptors admitted to rescue centres in the Czech Republic in the period from 2010 to 2019 by species.

Species		Number of admitted birds		IUCN Red list	Conservation status in the Czech Republic
Common name	Latin name	Number	%		
Common kestrel	*Falco tinnunculus*	13,019	57.76^a^	LC	NotT
Common buzzard	*Buteo buteo*	5,757	25.54^b^	LC	NotT
Eurasian sparrowhawk	*Accipiter nisus*	1,898	8.42^c^	LC	E
Northern goshawk	*Accipiter gentilis*	598	2.65^d^	LC	T
Marsh harrier	*Circus aeruginosus*	574	2.55^d^	LC	T
Honey buzzard	*Pernis apivorus*	138	0.61^e^	LC	E
Montagu’s harrier	*Circus pygargus*	97	0.43^f^	LC	E
White-tailed eagle	*Haliaeetus albicilla*	94	0.42^f,g^	LC	CE
Eurasian hobby	*Falco subbuteo*	69	0.31^g,h^	LC	E
Peregrine falcon	*Falco peregrinus*	63	0.28^h^	LC	CE
Red kite	*Milvus milvus*	60	0.27^h^	LC	CE
Other	*	171	0.76		
Total		22,538	100		

^a-h^ the values with different superscript letters in a column are statistically significantly different (p < 0.05).

LC = least concern; CE = critically endangered; E = endangered; T = threatened; NotT = not threatened.

*Other species: *Falco columbarius*, *Parabuteo unicinctus*, *Buteo lagopus*, *Buteo jamaicensis*, *Milvus migrans*, *Circus cyaneus*, *Aquila heliaca*, *Clanga pomarina*, *Aquila chrysaetos*, *Pandion haliaetus*, *Falco sparverius*, *Falco vespertinus*, *Falco rusticolus*, *Falco cherrug*, *Falco peregrinus pelegrinoides*.

**Fig 2:**
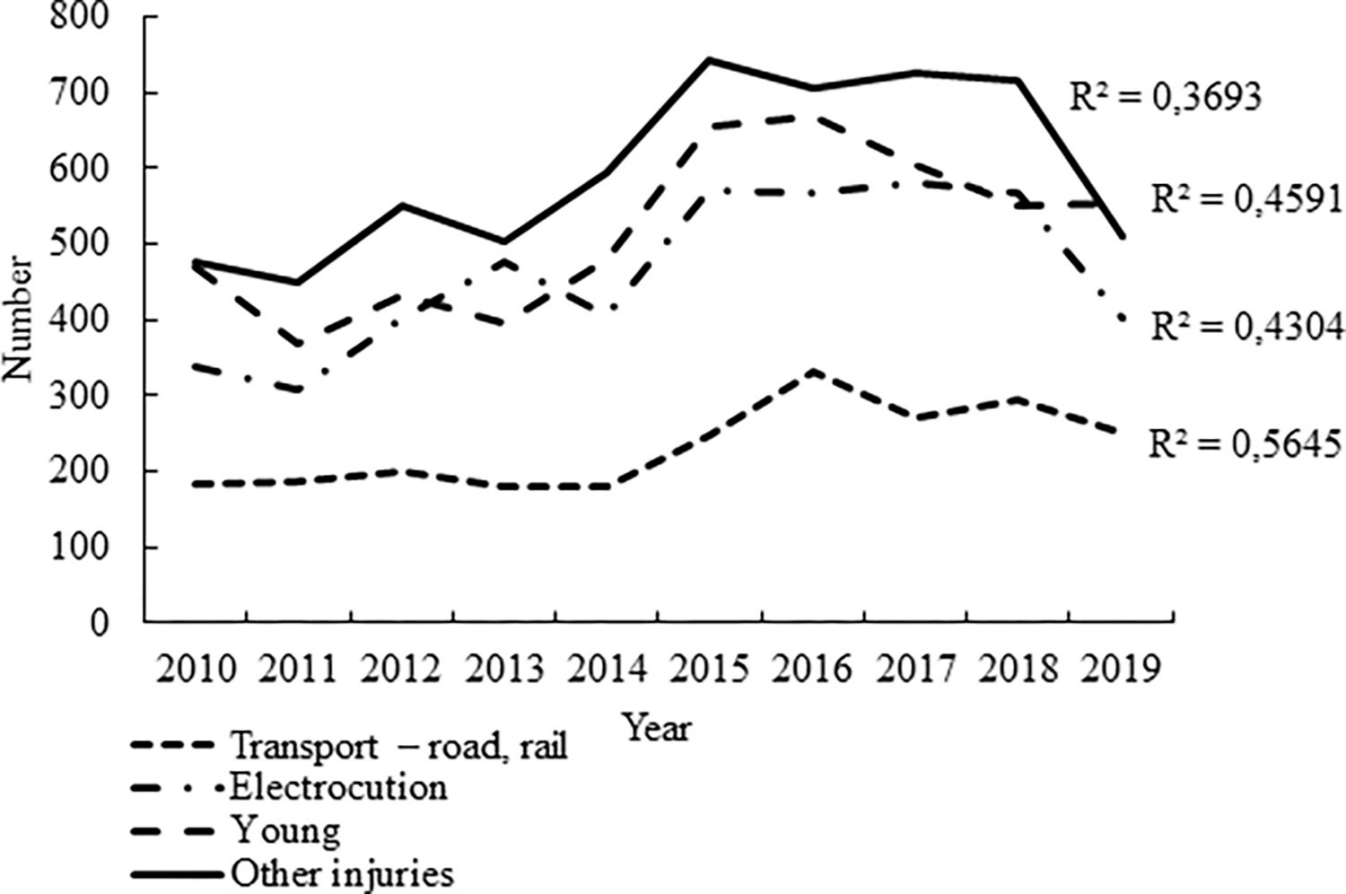
Numbers of raptors admitted to rescue centres in the Czech Republic in the period from 2010 to 2019 for the most common reasons for admission.

The most frequent reasons for the admission (Table 4) of adult birds were other injuries (26.52%) and electric shock injuries (20.51%). The admission of young and admission of raptors injured by road or trail vehicles was also frequent (22.98% and 10.34%, respectively). An increasing trend in numbers of admitted birds during the monitored period (rSp > 0,6; p < 0.05) was found for all four most common reasons for admission (Fig 2).

**Table 4:** Number and percentage of raptors admitted to rescue centres in the Czech Republic according to the reasons for their admission.

Reason for admission	Raptors admitted	
	(n = 22,538)	
	Number	%
**Other injuries**	5,976	26.52^a^
**Young**	5,179	22.98^b^
**Electric shock injuries**	4,623	20.51^c^
**Transport–road, rail**	2,331	10.34^d^
**Other**	1,825	8.10^e^
**Exhaustion and infection**	1,666	7.39^e^
**Inadvertent or unnecessary capture**	227	1.01^f^
**Falls into chimneys, etc.**	218	0.97^f^
**Hatchling**	168	0.75^g^
**Trapped or entangled**	125	0.55^h^
**Trained raptors**	106	0.47^h,i^
**Poisoned**	94	0.42^i,j^
**Total**	22,538	100

^a-j^ the values with different superscript letters in a column are statistically significantly different (p < 0.05).

Considering the most frequently admitted raptor species, differences (p < 0.05) were found among the most common reasons for their admission (Table 5). Whereas common kestrels were most often admitted as young, common buzzards, Eurasian sparrowhawks, northern goshawk, and marsh harriers were most frequently admitted due to other injuries.

**Table 5:** Number of birds admitted for selected (most frequent) reasons in the most common raptor species.

Species	Number of raptors admitted for individual reasons							
	Electric shock injuries		Other injuries		Young		Transport—road, trail	
	number	%	number	%	number	%	number	%
Common kestrel	3,434	26.38^a^	2,415	18.55^a^	4,488	34.47^a^	617	4.92^a^
Common buzzard	1,003	17.42^b^	1,683	29.23^b^	345	5.99^b^	1,368	23.76^b^
Eurasian sparrowhawk	54	2.85^c^	1,234	65.02^c^	104	5.48^c^	193	10.17^c^
Northern goshawk	23	3.85^d^	221	36.96^d^	24	4.01^d^	44	7.36^d^
Marsh harrier	39	6.79^c,d^	252	43.90^d^	102	17.77^c^	49	8.54^d^

^a-d^ the values with different superscript letters in a column are statistically significantly different (p < 0.05).

The outcomes for raptors admitted to rescue centres are given in Table 6. It proved possible to release 42.45% of admitted raptors back into the wild. Of them, 91.05% of raptors were released using the hard-release method. The soft-release method was used less often. More than a third (39.97%) of admitted raptors died or had to be euthanized at rescue centres. Considering the most common reasons for admission, the highest mortality rate was found in birds admitted due to electric shock injuries (81.25% of admitted raptors died or had to be euthanized). In contrast, only 5.81% of admitted young died or had to be euthanized (Table 7).

**Table 6:** The number and percentage of raptors admitted to rescue centres in the Czech Republic in the period from 2010 to 2019 according to the outcome.

Outcome	Raptors admitted	
	n = 22,538	
	number	%
**Death, euthanasia**	9,009	39.97^a^
**Hard—release**	8,711	38.65^b^
**Under continuing treatment**	1,530	6.79^g^
**Permanent captivity**	1,319	5.85^d^
**Other**	798	3.54^e^
**Soft-release**	587	2.60^f^
**Released without going into captivity**	255	1.13^g^
**The use of replacement nest**	225	1.00^g^
**Returned to owner**	59	0.26^h^
**Adoption**	45	0.20^h^
**Total**	22,538	100

^a-h^ the values with different superscript letters in a column are statistically significantly different (p < 0.05).

**Table 7:** The number and percentage of raptors that died or had to be euthanized at rescue centres in the Czech Republic in the period from 2010 to 2019 for the main reasons for admission.

Reason for admission	Number of admitted raptors for the given reason	Mortality rate (number of dead or euthanized birds)	
		number	%
**Transport–road, rail**	2,331	1,284	55.08
**Electric shock injuries**	4,623	3,756	81.25
**Young**	5,179	301	5.81
**Other injuries**	5,976	2,510	42.00

**Fig 3:**
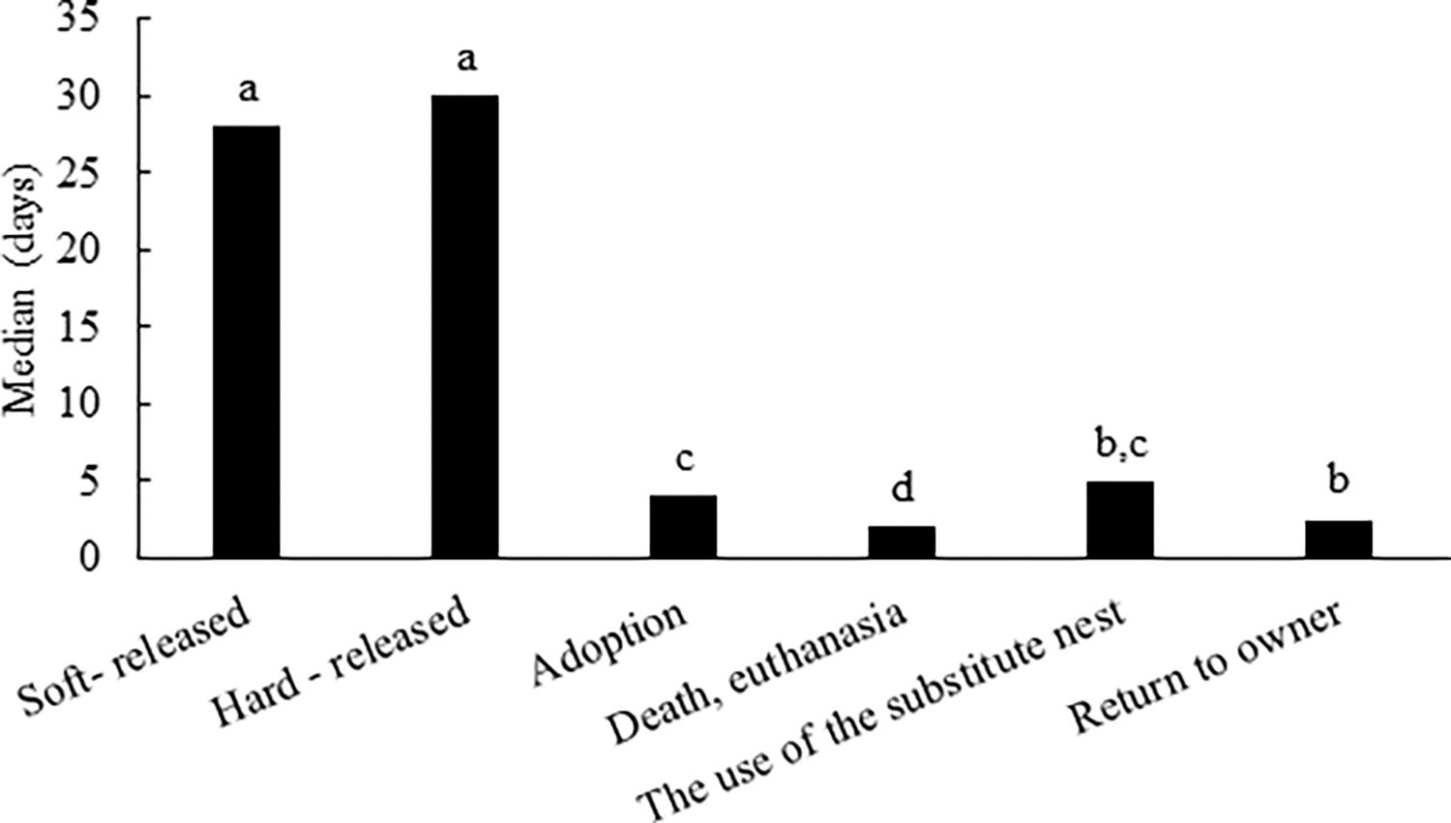
The length of time spent by raptors at rescue centres in the Czech Republic by outcome. ^a-d^ The values in columns with different superscript letters are significantly different (p < 0.05).

The length of time spent at rescue centres differed (p < 0.05) in raptors with various outcomes (Fig 3). The raptors that were subsequently released using the hard-release method (median 30 days, range 1–1,442 days) and the soft-release method (median 28 days, range 1–151 days) spent the longest time at rescue centres. The length of time there did not affect release type (p ˃ 0.05). The raptors that died or had to be euthanized at rescue centres spent usually only a couple of days there (median 2 days, range 1–2,358 days).

## Discussion

### Number of raptors admitted to rescue centres

Many countries have established rescue centres to provide care for injured animals belonging to wild species in the given area. Some countries have rescue centres specialising in certain groups of animals, such as raptors [23, 24]. In the Czech Republic, rescue centres may not decide to admit only selected species, as the national legislation obliges these facilities to admit all individuals of all species of wild animals that require their help. The increasing numbers of raptors admitted to rescue centres in the Czech Republic from 2010 to 2019 may not only testify to a higher level of risk for these animals in the wild, often associated with anthropogenic activity [25], but may also be a result of greater interest from the public. The people who find an injured raptor are usually those who bring these birds to rescue centres or contact the staff of rescue centres who then capture the given animal. Rescue centres play an important role in the protection of raptors, particularly with a view to the fact that the populations of these species are declining in the wild [26]. The raptor species admitted most often in the Czech Republic in the period from 2010 to 2019 were the common kestrel and the common buzzard. The species composition of raptors admitted to rescue centres may differ depending on which species live in a given area but some species may also be more threatened by anthropogenic activity [22]. Kestrels and buzzards are the most common species of raptor in the Czech Republic, and their larger numbers at rescue centres may correspond to this. In contrast, the red kite and the peregrine falcon, for example, are endangered species in the wild in the Czech Republic, and the lower numbers of red kites and peregrines admitted probably reflect their lower density in the Czech Republic rather than the fact that these are species that are affected to a lesser extent.

### Reasons for admission of raptors to rescue centres

The most frequent reason for the admission of raptors to rescue centres in the Czech Republic was other injury (i.e. injury other than electric shock or transport). In view of the fact that the lives of animals in the wild generally go unobserved, it is extremely difficult to determine the causes of these injuries without a thorough knowledge of the circumstances involved in each particular case. Injuries may be caused by another raptor since it is not uncommon for certain species of raptor to attack other raptors when hunting [27]. Cases in which a raptor is injured by a road or rail vehicle can usually be determined, as a raptor injured in this way would be found in the vicinity of a road or a rail track. In the Czech Republic, 10.34% of raptors were admitted due to their collision with a vehicle; however, Maphalala [28] in South Africa, for example, reported a higher number, in their study, 52% (242) of the raptors were admitted after collision with a vehicle. The risk of collisions between raptors and motor vehicles is increased by the fact that some species of raptors feed on the carcasses of animals hit by motor vehicles on highways [29]. Injuries were also reported among the most frequent reasons for the admission of raptors to rescue centres in other studies [30]. Traumatic injuries, not further specified, were the reason for the treatment of 75.80% (402) of raptors admitted to a rescue centre in Greece in the years 1997–2000 [31] and 49% (7,021) of raptors admitted to a rescue centre in Spain [32]. In addition to collisions with motor vehicles, other causes leading to injury and arising from human activity, such as gunshot wounds, were also reported [33].

The electric shock injuries of raptors, the third most common cause of admission of raptors to rescue centres in the Czech Republic, is more frequent than in other bird species [34]. Electricity pylons and power lines comprise an extremely dense network, particularly in some countries including the Czech Republic. Raptors may use them when they are searching for prey, observing the surrounding area or resting. Certain species were found to be electrocuted more often than others [35], probably as the result of specific morphological characteristics of certain species, such as wing length, overall length and tail length [36]. An electric current may cause moderate or severe injuries that cause either an immediate death of the raptor or an injury that the raptor is capable of surviving for a limited time. Even when birds are not killed immediately, they often die as a result of their inability to search for food or hunt. The technical design of electric power lines also presents a problem, in addition to the morphological body structure of raptors [37, 38]. Modification of electricity pylons may lead to a reduction in the mortality of raptors in the wild and prevent their injury [39].

### The release rate of raptors in rescue centres

Results of previous research studies show that raptors are successfully treated at rescue centres and returned to the wild in around fifty per cent of cases. In Alabama, in the period 2010–2014, 38% of raptors admitted to a rescue centre were released [40]. In the Czech Republic, 42.45% of raptors admitted to rescue centres were released. A release rate even higher was reported by a rescue centre in Jordan, 55.8% of admitted raptors were subsequently released there [41]. Birds that have recovered may be released from rescue centres in different ways. Although the methods of release may vary to some extent from one rescue centre to another, the National Network of Rescue Centres, which is responsible for the activities of all Czech rescue centres, apply measures to unify procedures throughout the country via training and uniform guidelines. In cases in which young are taken from their parents, a rapid response can make it possible to return such young to the nest after an evaluation of their health–there is a good chance in such cases that the young will be accepted by their parents [42]. Adoption by foster parents is rare, though not impossible. Even a successful adoption of a young by adults of a different raptor species has been reported [43]. In cases involving a mother with young that have found themselves in a dangerous place or whose nest has been destroyed, it is also possible to use a replacement nest and observe this nest to see whether the mother abandons it or not.

Various release methods are used in adult individuals, one of them being soft release, in which the animal is provided with food and shelter that it can use for a certain period after being released. Hard release, in contrast, involves individuals being released without further support. The method of release likely affects the chances of survival of the released individual. Mitchell et al. [44] and Tetzlaff et al. [45] demonstrated a greater survival rate in soft-released owls. According to several studies, the soft-release method of releasing birds back into the wild leads to a greater chance of survival thanks to gradual acclimatisation and the acquisition of sufficient energy reserves, while other positive aspects include the chance of observing the birds and their health status [46]. This release method is, nevertheless, used far less often in the Czech Republic than the hard-release method, which was used in 91.05% of all raptors released. Hard-release is recommended in older birds that have already been capable of looking after themselves completely before their time spent at a rescue centre.

If a raptor is not released directly from the rescue centre, it is appropriate to select a new release site as similar as possible to the site at which the bird was found especially for territorial species or the breeding season. However, it should be a place with no power lines nearby. It is important that the area is safe and that it has sources of food and other individuals of the same species. In view of the fact that raptors have not been further monitored after being released from the rescue centres included in our study, there is no way of determining whether the choice of the release method had an impact on their further survival or whether the preference for the hard-release method that predominated significantly in the Czech Republic can be recommended. One method of tracking the fate of raptors after they have been released from rescue centres, for example, is the use of telemetry [47].

### Length of stay of raptors in rescue centres

The length of time spent by raptors at rescue centres in the Czech Republic was comparable with the length of time spent by raptors at rescue centres in Greece [31]. Raptors that could be subsequently released back into the wild spent around a month at rescue centres. Death or euthanasia usually occurred shortly after admission. This testifies on one hand to the serious condition of these individuals on their admission to the rescue centre, and on the other hand to rapid decision-making in the case of euthanasia when their state of health is assessed. The fact that individuals with untreatable injuries were euthanized immediately without being exposed to long-lasting efforts at a futile treatment that would, in any case, lead to their death or euthanasia can be considered positive from the viewpoint of their welfare. Immediate euthanasia of terminal cases also directs the majority of limited resources to the birds who will benefit from it, and avoids wasting resources on birds who will not benefit. The raptor mortality at rescue centres in the Czech Republic (39.97% of raptors admitted) was comparable with the number of raptor deaths at a rescue centre in Iowa, at which 41% of admitted raptors died or had to be euthanized in the period 1986–1987 [33]. It can be assumed that the length of stay is also influenced by the condition of the individual on admission, with some injuries requiring not only surgery but also varying lengths of reconvalescence. The failure or success of care provided by a rescue centre does not depend merely on whether the given individual survives or dies, but primarily on whether it is possible to release it back into the wild or not, for which reason great attention is devoted to care for raptors and, in the case of young for example, great emphasis must be placed on preventing imprinting on humans [48]. The quality of life of birds after their release from rescue centres can be monitored by, e.g, telemetry and recording the position of the raptor using GPS navigation [49]. Such methods can help determine whether different release methods or techniques used in the care provided at rescue centres have differing impacts on these animals.

## Conclusion

Rescue centres help return injured or otherwise debilitated raptors and their young to the wild, thereby alleviating the impacts of anthropogenic activities on these animals. Data obtained from rescue centres may reflect the causes of the threats to raptors in the wild to a certain extent and may be used in the detection and mitigation of such threats, as in the case of, e.g., electric power lines. Further research focusing on care for raptors at rescue centres may contribute towards perfecting treatment and ensuring greater success in treating and subsequently releasing these individuals. According to the results of our study and other studies, the release rates are at a level of around 50%. Further research must, however, also be directed at monitoring survival rates in raptors released back into the wild and the possible effect of release methods, using telemetry for example.
